# Supplementary material for: Education and Training of Non-Genetics Providers on the Return of Genome Sequencing Results in a NICU Setting
Source: J Pers Med. 2022 Mar 5;12(3):405. doi: 10.3390/jpm12030405 (PMC8949881; doi:10.3390/jpm12030405)
Supplement: Supplementary file 1 [file jpm-12-00405-s001.zip › jpm-1548405- Suppl-S1 and S2.pdf]

Journal of *Personalized Medicine*

Supplementary Materials

S1: SouthSeq Provider Training Schedule

SouthSeq Provider Live Training

1. Purpose

Equip healthcare providers with the knowledge and skills needed to be able to understand genome sequencing results and discuss results with patients and families in a Neonatal Intensive Care Unit (NICU) setting.

2. Description

Live training of participating SouthSeq healthcare providers will occur at each study site and will last approximately 4 hours in total. The training will incorporate didactic learning, hands-on learning, and small group discussions to achieve the desired learning objectives. In addition to live training, materials from the training will be stored in the online Genome Gateway education platform for providers to review as needed throughout the study.

3. Learning Objectives

1. Explain the benefits and limitations of genome sequencing and how it compares to other types of genetic tests;
2. State the purpose of the SouthSeq study and the hypothesis being tested through result disclosure;
3. Identify the role of the non-genetics NICU provider in the SouthSeq study;
4. Demonstrate familiarity and proficiency completing provider tasks in the online Genome Gateway platform;
5. Interpret a SouthSeq genome sequencing result letter and report;
6. Develop a plan for disclosing various types of genome sequencing results (positive, negative, uncertain) including key points and next steps;
7. Describe common questions among patients receiving genome sequencing results;
8. Attend to psychosocial needs of families surrounding genome sequencing result disclosure;
9. Identify and critique patient support resources relevant to genome sequencing results.

4. Draft Schedule

15 minutes	Meet and greet, introductions
15 minutes	Overview of the SouthSeq study (Didactic)
15 minutes	Genome sequencing (Didactic)
15 minutes	Logistics of return of results in SouthSeq (Didactic)
10 minutes	Questions and answers
15 minutes	Logistics of the trial (Didactic)
45 minutes	Genome Gateway training (Hands-on)
60 minutes	Returning WGS results (Hands-on, small group discussion)
60 minutes	Anticipating impact (Didactic, simulation)
30 minutes	Wrap up, questions and answers

5. Description of Non-Didactic Portions of Training

5.1. Genome Gateway Training

Providers will use their own device (tablet, laptop) or a provided device to log into the Genome Gateway system. They will be led through a series of step by step instructions to learn how to 1) review patient information, 2) view files, 3) review educational materials, and 4) answer questionnaires.

5.2. Returning WGS Results

This session will begin with a brief didactic overview of what SouthSeq results look like. Next, providers will be given six example reports to review. As they review these reports, they will be asked to answer questions about each result on a written table. The goal of this activity is to become familiar with reports and know where to find information. Following this activity, attendees will break into smaller groups for a discussion facilitated by a genetic counselor. Genetic counselors will have a discussion guide to scaffold conversations, highlighting important take home messages and nuances of each example result.

5.3. Anticipating Impact

This session will begin with a brief didactic presentation, where genetic counselors will share their experiences giving genome results to patients and the possible range of responses and questions. We will also discuss strategies for assessing patient understanding and avoiding information overload when presenting genomic information. The final activity of the training includes a one-on-one simulation between each participating provider and a genetic counselor. The provider will be given the opportunity to practice describing an example result to the genetic counselor as well as responding to some typical (and not so typical) patient questions.

S2. SouthSeq Provider Training Survey Instruments

Pre-Training Survey

1. What is your primary role as a healthcare provider?

☐ Physician (MD/DO)
☐ Nurse practitioner (APRN/MSN/DNP)
☐ Physician's Assistant (PA-C)
☐ Genetic counselor
☐ Other______________

2. What is your specialty? (select all that apply)

☐ General Internal Medicine
☐ Internal Medicine Subspecialty______________
☐ General Pediatrics
☐ Pediatrics Subspecialty______________
☐ Family practice
☐ General Med/Peds
☐ Med/Peds Subspecialty
☐ Neurology
☐ Ob/Gyn
☐ General Surgery
☐ Surgery Subspecialty______________
☐ Psychiatry
☐ Hem/Onc
☐ Medical Genetics
☐ Other______________

3. Have you received any of the following formal genetics education (not including college or professional degree)? (select all that apply)

☐ Genetics residency or fellowship
☐ Genetics education course/CME (online or in person)
☐ Residency rotation in genetics
☐ Graduate degree (in addition to your professional degree) focused on genetics
☐ Other
☐ I have had no formal genetics training.

4. How many years have you been in practice (since residency/training ended)?

☐ 0-5 years
☐ 6–10 years
☐ 11–15 years
☐ 16–20 years
☐ 21–25 years
☐ >25 years

5. Please pick the category that best describes your race/ethnicity: (select all that apply)

☐ American Indian or Alaska Native
☐ Asian
☐ Black or African American
☐ Hispanic or Latino
☐ Native Hawaiian or Other Pacific Islander
☐ White

6. Have often, on average, are you involved in ordering genetic testing (karyotype, microarray, gene sequencing, exome/genome sequencing) for a patient as part of your clinical practice? Pick the answer that best describes your experience.

☐ Every day
☐ Once per week
☐ Once per month
☐ Once per year
☐ I have never ordered a genetic test

7. Which of the following types of genetic test results have you ordered for a patient as part of your clinical practice? (select all that apply)

☐ Karyotype
☐ Microarray
☐ Single gene test or small panel test (<10 genes)
☐ Large gene panel test (>10 genes)
☐ Mitochondrial DNA testing
☐ Exome sequencing
☐ Genome sequencing
☐ Other_______________
☐ I have never ordered a genetic test

8. How confident do you feel in your ability to read and interpret genetic test results?

☐ Not at all confident
☐ A little confident
☐ Somewhat confident
☐ Very confident

9. Have you ever seen a whole genome sequencing result report for a patient as part of your clinical practice?

☐ Yes
☐ No
☐ Unsure

10. How confident do you feel in your ability to read and interpret a whole genome sequencing test result?

- ☐ Not at all confident
- ☐ A little confident
- ☐ Somewhat confident
- ☐ Very confident

11. How confident do you feel in your ability to explain a whole genome sequencing test result to a patient/family?

- ☐ Not at all confident
- ☐ A little confident
- ☐ Somewhat confident
- ☐ Very confident

12. How confident do you feel in your ability to manage a patient's care based on a whole genome sequencing result?

- ☐ Not at all confident
- ☐ A little confident
- ☐ Somewhat confident
- ☐ Very confident

13. Which of the following do you feel are significant barriers to the implementation of whole genome sequencing in routine NICU clinical care? (select all that apply)

- ☐ Test cost
- ☐ Lack of insurance coverage
- ☐ Limited diagnostic value
- ☐ Possibility of unexpected results
- ☐ Possibility of uncertain results
- ☐ Limited healthcare provider time
- ☐ Lack of healthcare provider knowledge/training
- ☐ Lack of patient understanding
- ☐ Turn around time
- ☐ Other_______________
- ☐ There are no barriers

Post-Training Survey

1. To what extent do you feel this training has increased your understanding of genomics and the role it can play in making a diagnosis?

☐ Not at all
☐ A little
☐ Somewhat
☐ Very

2. To what extent do you feel that this training has equipped you with the knowledge and skills needed to implement your role in the SouthSeq study?

☐ Not at all
☐ A little
☐ Somewhat
☐ Very

3. How confident do you feel in your ability to read and interpret a whole genome sequencing test result?

☐ Not at all confident
☐ A little confident
☐ Somewhat confident
☐ Very confident

4. How confident do you feel in your ability to explain a whole genome sequencing test result to a patient/family?

☐ Not at all confident
☐ A little confident
☐ Somewhat confident
☐ Very confident

5. How confident do you feel in your ability to manage a patient's care based on a whole genome sequencing result?

☐ Not at all confident
☐ A little confident
☐ Somewhat confident
☐ Very confident

6. What aspect of training was most valuable to you?______________
7. What aspect of training was least valuable to you?______________
8. Are there any topics that you wished had been covered, or covered more deeply? If so, what are they?______________
9. Do you feel that the format and structure of this training was appropriate for the topics (length, inclusion of discussion, hands-on, and simulation)?______________
10. How often do you expect to refer to the online version of training materials in the future?

- ☐ Not at all
- ☐ Rarely
- ☐ Sometimes
- ☐ Very often

11. Is there any other feedback you would like to provide about the training?________________

Example SouthSeq Result Letter

This letter describes genetic test results from the SouthSeq research study (research protocol No. 300000328).

Your child was enrolled because he or she had symptoms that may be due to a genetic problem.

Reason for testing: Based on information provided to the research lab, the child has a history of heart muscle changes and a family history of a sibling with similar symptoms.

Results related to the reason for testing (also called primary results):
Two genetic changes were found (also called a positive result)

Result

- The whole genome sequencing test found two changes in the *RPL3L* gene that together are likely the reason for most or all of your child's symptoms.
- Changes in this gene have been seen in people with dilated cardiomyopathy 2D (CMD2D).
- People with CMD2D can have changes to their heart muscle and heart failure.

Chance that family members could have the same genetic changes (recurrence risk)

- One of the genetic changes was found in his mother's blood. The other genetic change was found in his father's blood.
- *RPL3L* gene changes are thought to be recessive. This means that two gene changes together cause a problem.
- Having one genetic change in *RPL3L* does not cause CMD2D. One change means someone is a carrier for CMD2D.
- When two carriers of CMD2D have a child together, there is a 25% (1 in 4) chance with each pregnancy to have a child with CMD2D.

Future care

- Your child's doctors and nurses may talk with you about changes to your child's care based on this result.
- Other genetic tests may be needed for your child based on his personal and family medical histories.
- Please continue to follow-up with your child's healthcare providers to learn about new information, testing options, or research studies.
- Please see the end of this letter and the attached lab report for more specific information about these genetic changes.

Results NOT related to the reason for testing (also called secondary results):
No other genetic changes found (also called a negative result)

Keep in mind:

- In addition to looking for the reason for your child's symptoms, this test looked at 73 other genes that can cause disease in the future.
- The whole genome sequencing test did not find any specific genetic changes associated with risk of developing a disease in the future.
- This does not mean your child will not develop a genetic disease in the future. Humans have more than 20,000 genes. There are many gene changes that may cause disease that the lab cannot find or understand.

For More Information:

- About CMD2D: https://omim.org/entry/619371
- For support related to CMD2D: https://rarediseases.org/rare-diseases/pediatric-cardiomyopathy/

If you have questions, please contact your child's healthcare team.
You may also contact a study genetic counselor at the information below.

About your child's variant(s).

Gene	Transcript	Variant
RPL3L	NM_005061.3	c.1076_1080del CCGTG (p.Ala359Glyfs*4)
RPL3L	NM_005061.3	c.80G>A (p.Gly27Asp)

The information in the table above is specific to your child. The "transcript" and "variant" describe the type and the specific location of your child's gene change.
